# Earth and life evolve together from something ancestral—reply to Britz *et al*.

**DOI:** 10.1098/rsbl.2022.0010

**Published:** 2022-03-23

**Authors:** Kazunori Yamahira, Shingo Fujimoto, Yasuoki Takami

**Affiliations:** ^1^ Tropical Biosphere Research Center, University of the Ryukyus, Okinawa 903-0213, Japan; ^2^ Graduate School of Human Development and Environment, Kobe University, Kobe 657-8501, Japan

**Keywords:** Gondwana supercontinent, *Lithopoecilus brouweri*, long-branch attraction, *Oryzias setnai*, ricefishes

## 1. Introduction

Britz *et al*. [1] objected to our conclusion that ricefishes of the family Adrianichthyidae dispersed eastward ‘out-of-India’ after the collision of the Indian subcontinent with Eurasia and subsequently diversified in Southeast-East Asia [2], based mainly on the following three points: (i) artefacts involved in ancestral area reconstruction by BioGeoBEARS in RASP [3], (ii) uncertainty of the phylogenetic position of *Oryzias setnai* because of long-branch attraction (LBA) and (iii) inadequate calibration using the fossil species †*Lithopoecilus brouweri*. Our replies to these three points are as follows.

## 2. Artefact in ancestral area reconstruction

First, we were aware that short trees lead to inaccurate maximum-likelihood estimates in BioGeoBEARS (http://phylo.wikidot.com/biogeobears-mistakes-to-avoid#toc2). This was likely the case for the tree used in Yamahira *et al*. [2] because the estimated parameters and likelihoods were completely identical between DEC and DEC + J models without scaling up tree branches (see electronic supplementary material, table S1*a* and *b*). Indeed, likelihoods also did not differ between the two models in most of the analyses in [1]. However, we considered that this may be a bug in RASP (v. 4.2); re-analysis with the original BioGeoBEARS (v. 1.1.2) [4–6] generated reasonable parameter estimates and likelihoods without scaling up branch lengths. This re-analysis (electronic supplementary material, table S1*c*) supported the DEC + J model (unlike in [1]) but estimated that the most recent common ancestor (MRCA) of Adrianichthyidae was distributed not only in the Indian subcontinent but also in Southeast Asia. We apologize and correct our previous result in [2].

**Figure 1.**
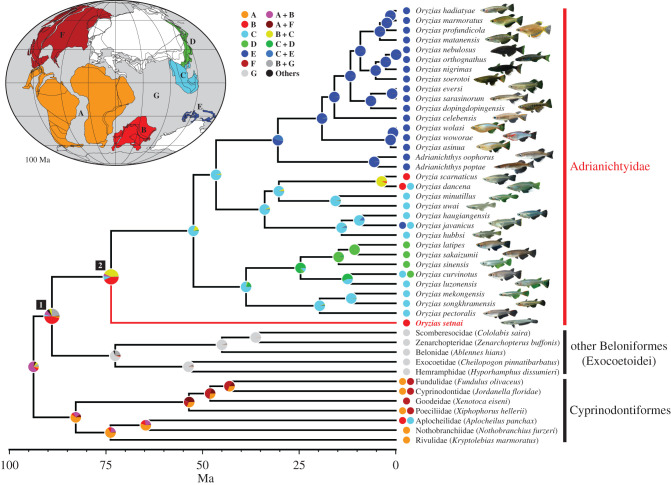
Ancestral areas at each node of the phylogenetic tree of Beloniformes and Cyprinodontiformes reconstructed under the DEC + J model by BioGeoBEARS.

Our interest then shifted to how this intercontinental distribution across the Tethys Sea was shaped. Because the cladogenesis of Cyprinodontiformes—the outgroup of Beloniformes to which Adrianichthyidae belongs—largely reflects the breakup of the supercontinent Gondwana in deep Mesozoic times [7–11], we conducted an additional BioGeoBEARS analysis, expanding the scope to include Cyprinodontiformes (see electronic supplementary material, supplementary material and methods and table S2 for details). The result revealed that an intercontinental distribution of the MRCA of Adrianichthyidae between India and Southeast Asia was not best supported; the most probable distribution area was on the Indian subcontinent only (node 2 in figure 1), and the MRCA of Beloniformes was also distributed only on the Indian subcontinent (node 1 in figure 1). These results strongly support the ‘out-of-India’ dispersal scenario.

Britz *et al*. [1] concluded that the fragmentation of a widely distributed coastal ancestral species better explains the historical biogeography of Adrianichthyidae. However, this ‘vicariance’ scenario is a premature conclusion derived from lack of consideration of the ancestral species' origin.

## 3. Phylogenetic position of *Oryzias setnai*

**Figure 2.**
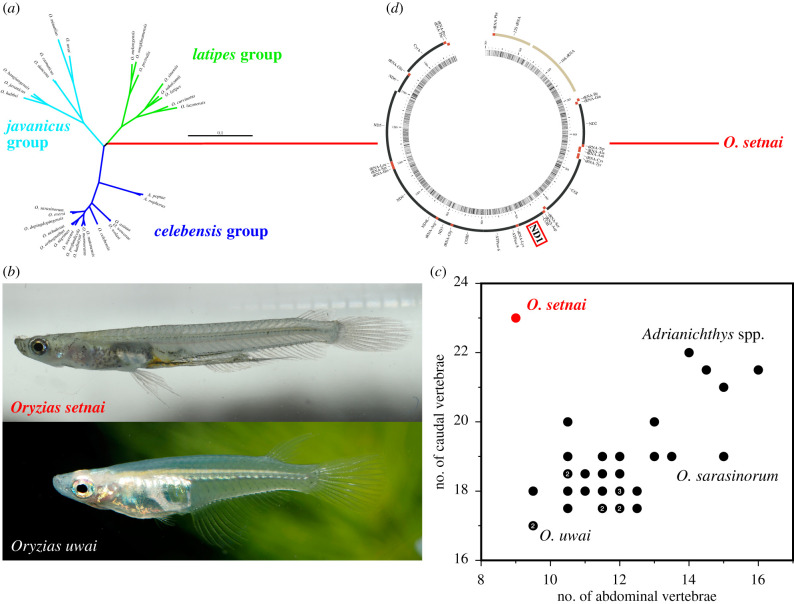
(*a*) Maximum-likelihood phylogeny of 33 adrianichthyid species based on mitochondrial (11 233 bp) and nuclear (4204 bp) sequences. We followed [1] except that all non-adrianichthyid outgroup species were excluded, and no outgroup was set. (*b*) Males of *Oryzias setnai* (photo by V. K. Anoop) and *O. uwai* (photo by N. Hashimoto). (*c*) Relationship between the number of abdominal and caudal vertebrae among 34 adrianichthyid species (see electronic supplementary material, table S3 for source references of raw data). Numbers within circles represent the number of species having the same combination of abdominal and caudal vertebral numbers. (*d*) Draft structure of the *O. setnai* mitochondrial genome. Note transposition of the ND1 gene downstream of the COI gene.

Second, we tested for the presence of LBA for *O. setnai* by estimating the phylogenetic position of this species after removing all outgroups, as proposed by [12]. We found that the position of *O. setnai* did not change (i.e. the branch leading to *O. setnai* split off from the internal branch that separates the *latipes* group from others; figure 2*a*), indicating that the effect of LBA, if any, is not substantial. It is also clear that fig. 1 in [1] did not successfully resolve deep divergences among families and orders within Atherinomorpha; for example, the flyingfish *Cheilopogon pinnatibarbatus* (Beloniformes) and the silverside *Menidia menidia* (Atheriniformes) were close to each other, implying that the finding of *O. setnai* nested among other adrianichthyids is unreliable.

We were aware that Parenti [13] estimated from morphological comparisons that *O. setnai* is sister to *O. uwai*, so we respectfully correct our assertion to ‘no **molecular** study has investigated its phylogenetic position’. We agree with [13] in terms of the view that *O. setnai* is a member of adrianichthyids, for which monophyly is supported by 17 synapomorphic characters, such as the lack of vomer and rostral cartilage. However, we disagree that *O. setnai* is phylogenetically located within other adrianichthyids [1,13], because it is highly autapomorphic. It is the only adrianichthyid species having internal fertilization, with male anterior anal-fin rays modified into an intromittent organ (figure 2*b*), and a bilaterally asymmetric female body [13]. Moreover, the number of abdominal and caudal vertebrae of *O. setnai* is disproportionally uncommon compared with other adrianichthyids (figure 2*c*). We also reported in [2] that mitochondrial genome gene order differs from that typical of vertebrates including other adrianichthyids (figure 2*d*; see also electronic supplementary material, figure S1 in [2]). These highly autapomorphic traits of *O. setnai* are consistent with our phylogenetic estimation that this species is sister to all other adrianichthyids.

## 4. Usage of †*Lithopoecilus brouweri* as fossil calibration

Third, we included †*L. brouweri* in our fossil calibration [2] because Parenti [13] had classified this species within Adrianichthyidae, but we realized that this classification was only tentative. We therefore re-estimated the divergence time of *O. setnai*, excluding †*L. brouweri* from the calibration. Excluding this fossil species did not greatly affect the divergence time estimation for *O. setnai*; it was estimated to have diverged about 71 million years ago (Mya) (electronic supplementary material, figure S1), whereas it was 74 Mya in [2]. This outcome indicates that although we still think that †*L. brouweri* is the common ancestor of *O. sarasinorum* and *O. eversi* (extant species endemic to Sulawesi [14]), the divergence time estimation for *O. setnai* is independent of its authenticity.

## 5. Conclusion

In summary, the points raised by Britz *et al*. [1] do not undermine our conclusions about the origin and evolutionary history of Adrianichthyidae (i.e. an eastward ‘out-of-India’ dispersal). We emphasize that ‘earth and life evolve together from something more ancestral’.

## Data Availability

Data available from the Dryad Digital Repository: https://doi.org/10.5061/dryad.nvx0k6dtx [15].
